# Barriers and facilitators to anal cancer screening among people living with HIV in Puerto Rico

**DOI:** 10.1186/s12889-023-16847-6

**Published:** 2023-10-06

**Authors:** Gabriela Cruz, Jeslie M. Ramos-Cartagena, José L. Torres-Russe, Vivian Colón-López, Karen J. Ortiz-Ortiz, Luis Pericchi, Ashish A. Deshmukh, Ana Patricia Ortiz

**Affiliations:** 1 College of Public Health, University of South Florida, 13201 Bruce B Downs Blvd, Tampa, FL 33612 USA; 2 Medical Science Campus, University of Puerto Rico/MD Anderson Cancer Center Partnership for Excellence in Cancer Research, PO BOX 365067, San Juan, 00936-5067 Puerto Rico; 3 University of Puerto Rico Comprehensive Cancer Center, PO Box 363027, San Juan, 00936-3027 Puerto Rico; 4 Puerto Rico Central Cancer Registry, San Juan, Puerto Rico; 5 Department of Mathematics, University of Puerto Rico, Rio Piedras Campus, Medical Science Campus, PO BOX 365067, San Juan, 00936-5067 Puerto Rico; 6 Medical University of South Carolina, 68 President St, BE 103, Charleston, SC 29425 USA; 7 Hollings Cancer Center, Medical University of South Carolina, Charleston, SC USA; 8 Graduate School of Public Health, Medical Sciences Campus, University of Puerto Rico, San Juan, Puerto Rico

**Keywords:** People living with HIV, Anal cancer screening, Anal Pap, High-resolution anoscopy, Barriers and facilitators

## Abstract

**Background:**

Anal cancer (AC) disproportionally affects people living with HIV (PLWH). Although there are no consensus-based AC screening guidelines, experts recommend anal pap as a primary screening tool in settings where high-resolution anoscopy (HRA) is available. We aimed to assess barriers and facilitators to anal cancer screening in a sample of Hispanic PLWH in Puerto Rico.

**Methods:**

To assess their knowledge and attitudes, we conducted a cross-sectional survey from 2020–2021 among PLWH in Puerto Rico (*n* = 212). Data was collected through a telephone interview that assessed information on sociodemographics, knowledge, and attitudes about AC, and the history of AC screening. The chi-square test, Fisher exact test, and logistic regression models were used to assess factors associated with screening uptake.

**Results:**

Anal Pap and HRA awareness were 60.4% and 30.7%, respectively. Anal Pap and HRA uptake was 51.5% and 19.3%, respectively. The most common barriers for anal Pap and HRA were lack of knowledge about the test and lack of physician recommendation. MSM were more likely to have heard of anal Pap (OR: 2.15, 95% CI:1.30–3.54) than MSW. MSM (OR: 3.04, 95% CI: 1.79–5.19) and women (OR: 3.00, 95% CI: 1.72–5.20) were also more likely to have undergone anal Pap. Similarly, individuals with a history of genital warts were more likely to have heard of anal Pap and HRA and have undergone anal Pap and HRA. Awareness of where to go for concerns about anal health was positively associated with having received anal Pap and HRA.

**Conclusions:**

With emerging evidence on the effectiveness of screening and treatment for anal cancer, several organizations are steering toward generating consensus-based anal cancer screening recommendations. Our study provides foundational data on barriers and facilitators to anal cancer screening in Puerto Rico that will be critical to informing screening implementation in this US territory.

## Background

Anal cancer incidence and mortality rates are continuing to rise at over 3% per year in the US [1]. Between 2001 and 2016, the rise of anal cancer among the general population in Puerto Rico has been rapid (Annual percent change [APC] = 4.9% and *P* = 0.0003) [2]. Anal cancer disproportionally affects certain high-risk populations such as people living with HIV (PLWH) [3]. In the United States, the incidence rate for anal cancer among PLWH is 19-fold greater when compared to the general population [4]. In Puerto Rico, it has been reported an incidence rate of anal cancer among PLWH of 27.7 per 100,000 person-years versus 1.1 per 100,00 person-years in the general population [2]. Persistent infection with high-risk human papillomavirus (HPV) is a necessary cause responsible for over 90% of anal cancer cases [5]. Moreover, Puerto Rico has a high prevalence of HIV (564 per 100,000 persons) and the HIV-attributable anal cancer burden in Puerto Rico (25% of the new SCCA cases in Puerto Rico were diagnosed in men living with HIV) is higher than that observed worldwide (20% of these new SCCA cases around the world were diagnosed in men living with HIV) [3, 6]. The increasing incidence of anal cancer and the high prevalence of HIV on the island highlights the importance of anal cancer screening among this high-risk population.

Despite the high burden of anal cancer in PLWH, evidence and consensus-based anal cancer screening guidelines have not yet been established. Expert groups recommend anal Pap test (cytology) as the primary screening tool among PLWH aged 35 years or older, but only in settings where high-resolution anoscopy (HRA), the gold standard diagnostic tool for high-grade squamous intraepithelial lesions (HSIL), is available [7–9]. The rationale for screening is to detect HSIL, the precursor lesions of anal cancer, and refer patients for treatment to prevent progression to cancer. Professional organizations such as the New York State Department of Health AIDS Institute and the HIV Medicine Association of the Infectious Disease Society of America recommend anal cancer screening for PLWH. Recently, the ANCHOR study reported that treatment of anal HSIL in PLWH decreased the incidence of anal cancer by half compared to those who did not receive treatment, a major milestone in understanding anal cancer prevention [10]. These findings serve as a catalyst to establish guidelines for anal cancer screening among PLWH, which are currently underway [11–13]. Given this development, there is an increased emphasis on studying implementation aspects of anal cancer screening, including barriers and facilitators, to facilitate the adoption and sustainment of emerging anal cancer screening recommendations.

Barriers and facilitators for anal cancer screening can be categorized into three levels: patient, provider, and system [14]. At the patient level, knowledge/awareness that they need to screen for anal cancer is a major barrier. The most commonly identified barrier at the provider level is the lack of scientific evidence for providers to determine what screening methods to implement for their patients [15–17]. Capacity issues (lack of providers trained to perform HRA) represent a systems-level barrier, where patients express waiting for several months for their appointments, which is a barrier to their HRA follow-up care [18, 19].

Facilitators at the patient level can be described as having certain beliefs, attitudes, and sociodemographic factors. Similar to the barriers identified, healthcare providers' recommendations and general knowledge and awareness surrounding anal cancer served as strong facilitators for anal cancer screening [20, 21]. Provider-level facilitators should be observed given that patients express high levels of dependence and trust in their providers' recommendations and expertise on the matter [15, 20]. To adequately establish and implement screening guidelines, it is important to understand barriers and facilitators of anal cancer screening among PLWH. To our knowledge, this is the first study to identify barriers and facilitators for anal Pap and HRA uptake among PLWH living in Puerto Rico.

## Methods

A cross-sectional study was conducted in Puerto Rico from September 2020 to November 2021 among 212 PLWH aged 26 years or older. The study was promoted in immunologic clinics, radio, television, and social media (Facebook and Instagram). Interested participants called the research study at a centralized telephone number. After telephone-based informed consent, a telephone interview in Spanish was conducted with each participant using a structured interview questionnaire, which assessed information on demographics, clinical and lifestyle characteristics, as well as on knowledge and attitudes about anal cancer and screening. All telephone interviews were performed by trained research personnel. Eligible participants for this analysis had complete information on awareness and uptake of anal Pap and HRA (*n* = 202). Participants received a monetary incentive for their participation in the study.

The main outcome variables for this analysis were lifetime awareness and uptake of anal Pap and HRA. Awareness was defined as whether a participant had previously heard of the anal Pap or HRA procedures (yes/no). Uptake of anal Pap and HRA was defined as having undergone the corresponding test anytime during the lifetime (yes/no). Independent variables included questions regarding demographics (sex at birth, age, education level, annual income, marital status, medical history, health insurance, etc.), knowledge about anal cancer, knowledge about screening methods, and knowledge about risk factors. Questions were adapted from previous study questionnaires to determine the attitudes surrounding anal cancer such as believed susceptibility, severity, and overall concern for their health [22–24]. For participants who had undergone an anal Pap test and HRA, questions about what factors served as facilitators were asked. For participants who did not undergo anal Pap test and HRA, questions about what factors served as barriers were asked.

### Statistical analysis

Frequencies were measured to determine the sociodemographic and clinical characteristics of the study sample. Bivariate analyses using Chi-square and Fishers exact tests were performed to compare the sociodemographic characteristics and attitudes surrounding anal cancer with anal Pap and HRA awareness and uptake. Variables identified as relevant in the literature and/or significant in bivariate analysis (*p* < 0.05), were considered in multivariate analysis. Generalized linear models (GLM) were used to assess the relationship between age, education, income, genital warts history, and sexual risk group with the four anal screening metrics previously identified. In addition, a second GLM was done to assess the relationship between attitudes surrounding anal cancer and awareness and uptake of anal Pap and HRA. Interaction terms were assessed using the likelihood ratio test. We compared the logit, probit, and extreme value links and the results were consistent. All of them had comparable deviances (a measure of goodness of fit). However, the probit link is a good model all around, and yields estimates of odds lower or similar than the other two links (GLM probits shown in study results). All statistical analyses were conducted using Stata SE/17.0 software [25].

## Results

Among the study population, the median years living with HIV was 15 years (IQR, 9–25) and more than half were aged less than 55 years (53.5%). Individuals self-identified as either men (66.8%), women (32.2%), or transgender (1.0%). Almost half of the participants (43.8%) identified themselves as men who have sex with men (MSM). More than half of the sample had an income of less than $15,000 (66.7%) and had pursued education after high school (55.1%). Awareness of anal Pap (60.4%) and uptake of anal Pap (51.5%) were both more common than awareness of HRA (30.7%) and uptake of HRA (19.3%) (Table 1).

**Table 1:** Demographics and clinical characteristics of a sample of PLWH in Puerto Rico (*n* = 202)

Variable	n	Percent
Age in years (Median, IQR) 54 (46–58)		
< 55	108	53.5
≥ 55	94	46.5
Education		
More than high school	112	55.1
High school or less	90	44.9
Health Insurance^a^		
Public	169	84.1
Private	27	13.4
None	5	2.5
Annual Income^b^		
< $15,000	128	66.7
≥ $15,000	64	33.3
Marital Status		
Married/Living together	61	30.2
Single/divorced/separated/widowed	141	69.8
Sexual Orientation^c^		
Heterosexual	110	54.7
Homosexual	76	37.8
Bisexual	15	7.5
Sexual Risk Group^d^		
MSM	88	43.8
MSW	48	23.9
Women	65	32.3
Sex at Birth		
Male	137	67.8
Female	65	32.2
Gender Identity		
Man	135	66.8
Woman	65	32.2
Transgender	2	1.0
History of AIDS diagnosis		
Yes	41	20.3
No	161	79.7
Years living with HIV (Median, IQR) 15 years (9–25)		
BMI (kg/m^2^)		
Underweight	9	4.5
Healthy weight	62	30.7
Overweight	59	29.2
Obesity	72	35.6
Current Smoker		
Yes	60	29.7
No	142	70.3
Genital Warts history ^e^		
Yes	181	10.0
No	20	90.0
HPV Vaccination history		
Yes	16	7.9
No	186	92.1
Anal Pap awareness		
Yes	122	60.4
No	80	39.6
Anal Pap uptake		
Yes	104	51.5
No	98	48.5
HRA awareness		
Yes	62	30.7
No	140	69.3
HRA uptake		
Yes	39	19.3
No	163	80.7

Missing or unknown information (*n* = 1)^a^; (*n* = 10)^b^; (*n* = 1)^c^; (*n* = 1)^d^; (*n* = 1)^e^

**Fig. 1:**
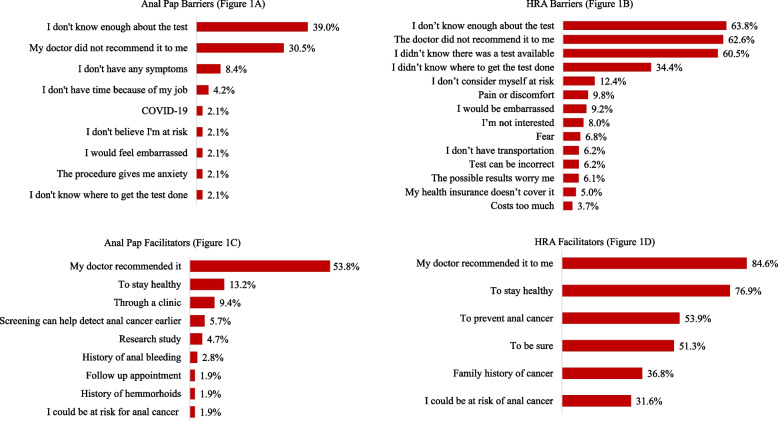
Barriers among those who never received Ana Pap or HRA and Facilitators among those who underwent Anal Pap or HRA, among a sample of PLWH in Puerto Rico

When analyzing barriers to anal Pap uptake, the three most common barriers included not knowing enough about the test (39.0%), lack of provider recommendation (30.5%), and not experiencing anal cancer symptoms (8.4%). Meanwhile, doctor recommendation (53.8%), to be healthy (13.2%) and that it was available in their immunology clinic (9.4%) were the top three facilitators for uptake of anal Pap (Fig. 1). For those who had undergone HRA, the three factors most reported as facilitators to HRA were doctor recommendation (84.6%), to stay healthy (76.9%), and to prevent anal cancer (53.9%). For those who had not undergone HRA, the three factors most identified as barriers were lack of knowledge about the test (63.8%), lack of doctor recommendation (62.6%), and lack of awareness of the availability of the test (60.5%) (Fig. 1).

In bivariate analysis, awareness of anal Pap was significantly associated with age, education, income, sexual orientation, genital warts history, and sexual risk group (*p* < 0.05). Whereas anal Pap test uptake was associated with education, sexual orientation, genital warts history, and sexual risk group (*p* < 0.05). Awareness and uptake of HRA were associated with genital warts history (Table 2). In addition, two attitudes were found to be significantly associated with anal Pap test uptake, “I know which doctor to go to if I am worried about my anal health” and “I would like to learn more about anal cancer” (*p* < 0.05). Meanwhile, for HRA uptake the following attitudes were statistically significant “I worry a lot about developing anal cancer” and “I know which doctor to go to if I am worried about my anal health” (*p* < 0.05) (Table 3).

**Table 2:** Factors associated with anal Pap and HRA awareness and uptake among a sample of PLWH in Puerto Rico (*n* = 202)

	**Anal Pap Awareness**			**Anal Pap Uptake**			**HRA Awareness**			**HRA Uptake**		
	Yes n (%)	No n (%)	*P* -Value	Yes n (%)	No n (%)	*P*—Value	Yes n (%)	No n (%)	*P*—Value	Yes n (%)	No n (%)	*P*—Value
Age (years)												
< 55	34 (31.5)	74 (68.5)	**0.011**	46 (42.6)	62 (57.4)	0.07	73 (67.6)	35 (32.4)	0.57	19 (17.6)	89 (82.4)	0.51
≥ 55	46 (48.9)	48 (51.1)		52 (55.3)	42 (44.7)		67 (71.3)	27 (28.7)		20 (21.3)	74 (78.7)	
Education												
More than high school	77 (68.8)	35 (31.2)	**0.007**	66 (58.9)	46 (41.1)	**0.018**	34 (30.4)	78 (69.6)	0.91	22 (19.6)	90 (80.4)	0.89
High school or less	45 (50.0)	45 (50.0)		38 (42.2)	52 (57.8)		28 (31.1)	62 (68.9)		17 (18.9)	73 (81.1)	
Health Insurance^a^												
Public	100 (59.2)	69 (40.8)	0.84*	86 (50.9)	83 (49.1)	1.0*	53 (31.4)	116 (69.0)	0.21*	34 (20.1)	135 (79.9)	0.84*
Private	18 (66.7)	9 (33.3)		14 (51.9)	13 (48.1)		6 (21.2)	21 (77.8)		4 (14.8)	23 (85.2)	
None	3 (60.0)	2 (40.0)		3 (60.0)	2 (40.0)		3 (60.0)	2 (40.0)		1 (20.0)	4 (80.0)	
Annual Income^b^												
< $15,000	72 (56.2)	56 (43.8)	**0.021**	67 (52.3)	61 (47.7)	0.76	43 (33.6)	85 (66.4)	0.32	28 (21.9)	100 (78.1)	0.20
≥ $15,000	47 (73.4)	17 (26.6)		35 (54.7)	29 (45.3)		17 (26.6)	47 (73.4)		9 (14.1)	55 (85.9)	
Marital Status												
Married/Living together	82 (58.2)	59 (41.8)	0.32	72 (51.1)	69 (48.9)	0.86	22 (36.1)	39 (63.9)	0.28	8 (13.1)	53 (86.9)	0.14
Single/divorced/separated/widowed	40 (65.6)	21 (34.4)		32 (52.5)	29 (47.5)		40 (28.4)	101 (71.6)		31 (22.0)	110 (78.0)	
Sexual Orientation^c^												
Heterosexual	52 (47.3)	58 (52.7)	**< 0.001**	44 (40.0)	66 (60.0)	**0.001**	29 (26.4)	81 (73.6)	0.30	20 (18.2)	90 (81.8)	0.89
Homosexual	59 (77.6)	17 (22.4)		52 (68.4)	24 (31.6)		28 (36.8)	48 (63.2)		16 (21.0)	60 (79.0)	
Bisexual	10 (66.7)	5 (33.3)		7 (46.7)	8 (53.3)		4 (26.7)	11 (73.3)		3 (20.0)	12 (80.0)	
Sexual Risk Group^d^												
MSM	66 (75.0)	22 (25.0)	**< 0.001**	56 (63.6)	32 (36.4)	**< 0.001**	31 (35.2)	57 (64.8)	0.13	18 (20.4)	70 (79.6)	0.35
MSW	18 (37.5)	30 (62.5)		9 (18.8)	39 (81.2)		9 (18.8)	39 (81.2)		6 (12.5)	42 (87.5)	
Women	37 (56.9)	28 (43.1)		38 (58.5)	27 (41.5)		21 (32.3)	44 (67.7)		15 (23.1)	50 (76.9)	
Sex at Birth												
Male	85 (62.0)	52 (38.0)	0.49	66 (48.2)	71 (51.8)	0.17	41 (29.9)	96 (70.1)	0.73	24 (17.5)	113 (82.5)	0.35
Female	37 (56.9)	28 (43.1)		38 (58.5)	27 (41.5)		21 (32.3)	44 (67.7)		15 (23.1)	50 (76.9)	
Gender Identity												
Man	82 (60.7)	53 (39.3)	0.60	64 (47.4)	71 (52.6)	0.18	41 (30.4)	94 (69.6)	0.94*	24 (17.8)	111 (82.2)	0.64*
Woman	38 (58.5)	27 (41.5)		39 (60.0)	26 (40.0)		21 (32.3)	44 (67.7)		15 (23.1)	50 (76.9)	
Transgender	2 (100.0)	0 (0.0)		1 (50.0)	1 (50.0)		0 (0.0)	2 (100.0)		0 (0.0)	2 (100.0)	
Diagnosed with AIDS												
Yes	25 (61.0)	16 (39.0)	0.93	21 (51.2)	20 (48.8)	0.97	13 (31.7)	28 (68.3)	0.88	7 (17.1)	34 (82.9)	0.69
No	97 (60.2)	64 (39.8)		83 (51.5)	78 (48.5)		49 (30.4)	112 (69.6)		32 (19.9)	129 (80.1)	
BMI												
Underweight	2 (22.2)	7 (77.8)	0.09	6 (66.7)	3 (33.3)	0.29	1 (11.1)	8 (88.9)	0.62	1 (11.1)	8 (88.9)	0.69
Healthy weight	36 (58.1)	26 (41.9)		26 (41.9)	36 (58.1)		20 (32.3)	42 (67.7)		12 (19.4)	50 (80.6)	
Overweight	39 (66.1)	20 (33.9)		32 (54.2)	27 (45.8)		19 (32.2)	40 (67.8)		14 (23.3)	46 (76.7)	
Obesity	45 (62.5)	27 (37.5)		40 (55.6)	32 (44.4)		22 (30.6)	50 (69.4)		12 (16.7)	60 (83.3)	
Current Smoker												
Yes	39 (65.0)	21 (35.0)	0.38	31 (51.7)	29 (48.3)	0.97	16 (26.7)	44 (73.3)	0.42	11 (18.3)	49 (81.7)	0.82
No	83 (58.4)	59 (41.6)		73 (51.4)	69 (48.6)		46 (31.4)	96 (67.6)		28 (19.7)	114 (80.3)	
Genital Warts history^e^												
Yes	19 (95.0)	1 (5.0)	**0.001**	17 (85.0)	3 (15.0)	**< 0.001**	12 (60.0)	8 (40.0)	**< 0.001**	9 (45.0)	11 (55.0)	0.005*
No	103 (56.9)	78 (43.1)		87 (48.1)	94 (51.9)		50 (27.6)	131 (72.3)		30 (16.6)	151 (83.4)	
HPV vaccination history												
Yes	9 (56.3)	7 (43.7)	0.724	9 (56.3)	7 (43.8)	0.691	6 (37.5)	10 (62.5)	0.576*	3 (18.9)	13 (81.3)	1.00*
No	113 (60.8)	73 (39.2)		95 (51.1)	91 (48.9)		56 (30.1)	130 (69.9)		36 (19.4)	150 (80.7)	

Missing or unknown information (*n* = 1)^a^; (*n* = 10)^b^; (*n* = 1)^c^; (*n* = 1)^d^; (*n* = 1)^e^

^*^Fisher exact test was used to assess association

**Table 3:** Attitudes around anal cancer and history of anal Pap and HRA among a sample of PLWH in Puerto Rico (*n* = 202)

	**Anal Pap Uptake**			**HRA Uptake**		
	Yes n (%)	No n (%)	*P*—Value	Yes n (%)	No n (%)	*P*—Value
“I have a greater chance of developing anal cancer”^a^						
Agree	58 (59.2)	40 (40.8)	0.21	24 (66.7)	12 (33.3)	0.11
Disagree	45 (50.0)	45 (50.0)		79 (52.0)	73 (48.0)	
“I worry a lot about developing anal cancer”^b^						
Agree	89 (86.4)	14 (13.6)	0.07	37 (94.9)	2 (5.1)	**0.017**
Disagree	74 (76.3)	23 (23.7)		126 (78.3)	35 (21.7)	
“Anal cancer is a hopeless disease”^c^						
Agree	25 (25.2)	74 (74.8)	0.45	7 (18.4)	31 (81.6)	0.16
Disagree	28 (30.1)	65 (69.9)		46 (29.9)	108 (70.1)	
“I’m scared of being diagnosed with anal cancer”^d^						
Agree	82 (79.6)	21 (20.4)	0.23	30 (79.0)	8 (21.0)	0.65
Disagree	71 (72.5)	27 (27.5)		123 (75.5)	40 (24.5)	
“Early detection methods for anal cancer and very uncomfortable”^e^						
Agree	48 (49.0)	50 (51.0)	0.28	23 (62.2)	14 (37.8)	0.17
Disagree	33 (57.9)	24 (42.1)		58 (49.2)	60 (50.8)	
“I know which doctor to go to if I am worried about my anal health”^f^						
Agree	80 (77.7)	23 (22.3)	**0.003**	36 (92.3)	3 (7.7)	** < 0.001**
Disagree	51 (57.3)	38 (42.7)		95 (62.1)	58 (37.9)	
“I’m okay, I don’t have anal cancer, and therefore I am not interested in anal cancer screening”^g^						
Agree	14 (14.0)	86 (86.0)	0.09	3 (7.9)	35 (92.1)	0.059
Disagree	22 (23.4)	72 (76.6)		33 (21.1)	123 (78.9	
“Anal cancer has little effect on daily life”^h^						
Agree	19 (19.2)	80 (80.8)	0.83	9 (24.3)	28 (75.7)	0.32
Disagree	16 (18.0)	73 (82.0)		26 (17.2)	125 (82.8)	
“I would like to learn more about anal cancer”						
Agree	104 (100.0)	0 (0.0)	0.012*	38 (97.4)	1 (2.6)	1.0*
Disagree	92 (93.9)	6 (6.1)		158 (96.9)	5 (3.1)	

Missing or unknown information (*n* = 14)^a^; (*n* = 2)^b^; (*n* = 10)^c^; (*n* = 1)^d^; (*n* = 47)^e^; (*n* = 10)^f^; (*n* = 8)^g^; (*n* = 14)^h^

^*^Fisher exact test was used to assess association

In multivariate analysis, MSM (OR: 2.15, 95% CI: 1.30–3.54) had significantly higher odds of being aware of anal Pap test in comparison with men who have sex with women (MSW). Higher odds of being aware were also seen among individuals with a history of genital warts (OR: 4.58, 1.61–13.00). For HRA awareness, MSM (OR: 1.18, 95% CI: 1.01–1.39) and individuals with a history of genital warts (OR: 1.38, 1.12–1.70) had significantly higher odds than their counterparts (Table 4). Similarly, MSM (OR: 3.04 95% CI: 1.79–5.19) and women (OR: 3.00, 95% CI: 1.72–5.20) had significantly higher odds of having undergone an anal Pap test than MSW. Individuals with a history of genital warts were more likely to have undergone an anal Pap test (OR: 2.25 95% CI: 1.06–4.77) and HRA (OR: 1.96, 95% CI: 1.04–3.70) than their counterparts (Table 5).

**Table 4:** Generalized linear models^a^ of factors associated to anal Pap and HRA awareness among a sample of PLWH in Puerto Rico

	**Anal Pap Awareness** Crude OR (95% CI)	**Anal Pap Awareness** Adjusted OR (95% CI)	**HRA Awareness** Crude OR (95% CI)	**HRA Awareness** Adjusted OR (95% CI)
Age (years)				
< 55	1.0	1.0	1.0	1.0
≥ 55	**0.83 (0.73–0.96)**	0.71 (0.48–1.04)	0.96 (0.84–1.10)	0.99 (0.68–1.46)
Education				
High school or less	1.0	1.0	1.0	1.0
More than high school	**1.20 (1.06–1.38)**	1.47 (0.97–2.23)	0.99 (0.87–1.13)	0.91 (0.60–1.38)
Sexual Risk Group				
MSW	1.0	1.0	1.0	1.0
MSM	**1.45 (1.23–1.72)**	**2.15 (1.30–3.54)**	**1.18 (1.01–1.39)**	1.62 (0.96–2.75)
Women	**1.21 (1.02–1.45)**	1.62 (0.99–2.66)	1.15 (0.97–1.36)	1.43 (0.85–2.43)
Genital Warts history				
No	1.0	1.0	1.0	1.0
Yes	**1.46 (1.17–1.82)**	**4.58 (1.61–13.00)**	**1.38 (1.12–1.70)**	**2.23 (1.23–4.06)**

^a^Analyses using the probit link function

**Table 5:** Generalized linear models^a^ of factors associated with anal Pap and HRA uptake among a sample of PLWH in Puerto Rico

	**Anal Pap Uptake** Crude OR (95% CI)	**Anal Pap Uptake** Adjusted OR (95% CI)	**HRA Uptake** Crude OR (95% CI)	**HRA Uptake** Adjusted OR (95% CI)
Age (years)				
< 55	1.0	1.0	1.0	1.0
≥ 55	0.88 (0.77–1.01)	0.77 (0.52–1.15)	1.04 (0.93–1.16)	1.18 (1.53–5.24)
Sexual Risk Group				
MSW	1.0	1.0	1.0	1.0
MSM	**1.57 (1.33–1.85)**	**3.04 (1.79–5.19)**	1.08 (0.94–1.24)	1.14 (0.62–2.11)
Women	**1.49 (1.25–1.77)**	**3.00 (1.72–5.20)**	1.11 (0.96–1.29)	1.33 (0.71–2.51)
Genital Warts history				
No	1.0	1.0	1.0	1.0
Yes	**1.45 (1.15–1.81)**	**2.25 (1.06–4.77)**	**1.33 (1.11–1.59)**	**1.96 (1.04–3.70)**
“I worry a lot about developing anal cancer”	1.18 (0.99–1.41)	1.39 (0.82–2.36)	**1.19 (1.03–1.37)**	**2.46 (1.14–5.32)**
“I know which doctor to go to if I am worried about my anal health”	**1.26 (1.09–1.47)**	**1.92 (1.26–2.92)**	**1.25 (1.11–1.41)**	**2.83 (1.53–5.24)**

^a^Analyses using the probit link function

In addition, participants who agreed with the following statement, “I know which doctor to go to if I am worried about my anal health”, were twice more likely to have undergone an anal Pap test (OR: 1.92, 95% CI: 1.26–2.92) and HRA (OR: 2.83, 95% CI: 1.53–5.24) than those who did not agree with the statement. Similarly, participants who agreed with the following statement, “I worry a lot about developing anal cancer” were twice more likely to have undergone HRA (OR: 2.46, 95% CI: 1.14–5.32) in comparison to those who did not agree with the statement (Table 5).

## Discussion

This study serves to contribute to the existing literature regarding barriers and facilitators of anal cancer screening access in PLWH, with a particular focus on the Hispanic population living in Puerto Rico. Out of our total sample, 60.4% stated they had heard of the anal Pap test before, but only 30.7% of the sample had heard of HRA, which indicates to us that not all of the participants are receiving the same depth of information regarding screening methods.

The common theme across the top barriers for both anal Pap and HRA was a lack of knowledge surrounding these screening tests and a lack of physician recommendation to screen. Therefore, these results further underscore the need for standardized anal cancer screening guidelines for providers to have these discussions with their patients. Individual state guidelines have emerged, such as the one developed by the New York State Department of Health in conjunction with the AIDS Institute [9]. Also, efforts have been initiated, such as the advisory group on anal cancer screening and prevention under the STI guidelines, convened by the CDC in 2019 [8]. Both groups proposed anal cancer screening among high-risk populations starting at 35 years [8, 9]. Furthermore, organizations like the International Anal Neoplasia Society (IANS) strive to educate and promote the different screening measures as well as the standard use of the HRA. IANS also supports the training of physicians in HRA and has recommended minimum criteria for providers to be certified [26]. However, there is no universal public policy agreement that establishes screening guidelines for anal cancer across the nation. The lack of physician recommendations can be attributed to not only the lack of official guidelines for screening but also the low availability of trained physicians on screening methods and the lack of infrastructure.

Only 32% of those who underwent HRA stated they believed themselves to be at risk for anal cancer and only 54.8% correctly identified themselves as being at higher risk for developing anal cancer, our findings are congruent with previous studies that have found a generalized low perceived risk for anal cancer among high-risk populations [27–29]. This finding is important to note because these participants are demonstrating a dissonance between the actual and perceived risk of anal cancer which indicates that they may not seek screening methods due to their low perceived risk when in fact they have a higher risk for a persistent high-risk HPV infection and anal cancer occurrence.

When looking at the barriers to anal Pap test and HRA uptake selected by the participants, the most common barriers faced were knowledge or awareness of the test and the doctor’s recommendation. It is important to note that the barriers related to knowledge and awareness are referring to factors such as lack of awareness of the availability of the test, lack of knowledge of where to obtain the test, and simply not knowing enough about the test. Healthcare providers could address all these knowledge measures during clinical visits, providing an opportunity for them to help minimize multiple barriers to screening and diagnosis of anal cancer by providing education to their patients about the topic. Our results indicate that the vast majority of our participants were interested in learning more about anal cancer and those who expressed worry about developing anal cancer were more likely to have undergone an anal Pap test or HRA. In addition, this population not only wants to receive education about anal cancer screening methods but there was also a positive association between those who knew of a provider to go to if they had concerns about their anal health and uptake of screening and diagnostic methods.

On multivariate analysis, we found that MSM and women were more likely to have undergone an anal Pap test than MSW. Although MSM is the population at higher risk of anal cancer, women and MSW living with HIV are still at more high risk of developing anal cancer than their HIV-negative counterparts [30]. This result highlights the need for standardized screening guidelines targeting PLWH, and their proper implementation across all genders. In addition, individuals with a history of genital warts were more likely to have undergone anal Pap tests and HRA. This finding suggests that physicians are probably recommending more of these procedures to PLWH with genital warts. Although genital warts are caused by low-risk HPV types, individuals with anal warts are at increased risk of anal cancer [31–33]. We also found that, unlike previously published findings [34–36], that fear, embarrassment, and cost were not common barriers reported by the participants in our study. Nonetheless, the overall results of this study support previous findings that significant barriers to anal cancer screening exist at the patient, provider, and system levels of care [14, 15].

While other studies found lower rates of awareness and history of anal cancer screening than the ones reported in this population of Hispanic PLWH, those studies had slight methodological differences, such as looking at sub-populations that may face added barriers, such as women and transgender individuals [27–29, 37]. Acceptability of the screening methods has been previously established to be high among PLWH [35, 38, 39] and our findings support this with a vast majority of respondents (97%) being interested in learning more about anal cancer. We now know that based on evidence from the ANCHOR study, treatment for anal high-grade squamous lesions significantly lowered the incidence of anal cancer in PLWH [10], therefore guidelines for anal cancer screening have never been more appropriate. Results from the ANCHOR study support the use of anal cancer screening and HSIL treatment among PLWH that are 35 years or older. As we wait for guidelines to be implemented, research studies should focus on how to improve the patient-provider communication gap to not only educate on anal cancer but to also understand the unique needs the sub-populations of PLWH may have when discussing anal cancer risk [36].

### Strengths and limitations

To our knowledge, this is the first study in Puerto Rico to assess barriers and facilitators for anal cancer screening among PLWH. Furthermore, it is among the first to have been done in a Hispanic population, and to evaluate both anal Pap and HRA. Understanding barriers and facilitators to anal cancer screening methods is necessary to support the implementation of screening guidelines both at the systems and provider levels. This study is not without limitations. The information collected was self-reported and response bias can have a significant impact on the data analyzed. The sample size of this study was relatively small and given the limited uptake of the evaluated procedures, when analyzing the sub-samples of people who had undergone an anal Pap or HRA, sample sizes were limited. Finally, results are not generalizable to all PLWH in Puerto Rico. Nonetheless, despite these limitations, findings provide previously nonexistent information for this Hispanic population, which will aid the implementation of future anal cancer screening programs among PLWH.

## Conclusion

Physician recommendation, prevention of anal cancer, a desire to stay healthy, and accessibility were the most common facilitators for those who underwent an anal Pap or HRA. Lack of knowledge about the test, lack of doctor recommendation, lack of awareness of the availability of tests, and absence of symptoms associated with anal cancer were the most common barriers for those who did not undergo an anal Pap or HRA. A desire to learn more about anal cancer, concern about developing anal cancer, and fear of anal cancer diagnosis were highly reported attitudes among our sample. At the same time, approximately only half of the participants identified themselves as having a greater chance of developing anal cancer. This dissonance in actual and perceived risk is critical to understand when trying to engage PLWH in anal cancer screening. 

These findings help inform two primary recommendations for anal cancer screening implementation. The first recommendation is for the establishment of official anal cancer screening guidelines. Many physicians are unsure of anal cancer screening methods and their implementation because there are no designated guidelines for them to follow. Our second recommendation is that physicians or providers who work with PLWH need to educate themselves and their patients on topics such as HPV infection and the increased risk of HPV-related cancers in PLWH.

## Data Availability

The data that was used for the analysis of this study is available upon request from the corresponding author, (ana.ortiz7@upr.edu or jeslie.ramos@upr.edu). Is not publicly available due to containing information that could potentially compromise the privacy of the participants.

## Abbreviations

AP: Anal pap
PLWH: People living with HIV
HPV: High-risk human papilloma virus
HRA: High-resolution anoscopy
HSIL: High-grade squamous epithelial lesions
MSM: Men who have sex with men
